# *Chchd10* is dispensable for myogenesis but critical for adipose browning

**DOI:** 10.1186/s13619-022-00111-0

**Published:** 2022-04-01

**Authors:** Wei Xia, Jiamin Qiu, Ying Peng, Madigan M. Snyder, Lijie Gu, Kuilong Huang, Nanjian Luo, Feng Yue, Shihuan Kuang

**Affiliations:** 1 College of Animal Science and Technology, Hebei Agricultural University, Baoding, 071000 China; 2 Department of Animal Sciences, Purdue University, West Lafayette, IN 47907 USA; 3 College of Animal and Veterinary Science, Southwest Minzu University, Chengdu, 610041 China; 4 College of Biological Sciences, China Agricultural University, Beijing, 100193 China

**Keywords:** Skeletal muscle, Myoblasts, Regeneration, Adipocyte, Brown adipose tissue, Uncoupling protein 1 (UCP1)

## Abstract

The *Chchd10* gene encodes a coiled-coil-helix-coiled-coil-helix-domain containing protein predicted to function in the mitochondrion and nucleus. Mutations of *Chchd10* are associated with ALS, dementia and myopathy in humans and animal models, but how knockout of *Chchd10* (*Chchd10*^*KO*^) affects various tissues especially skeletal muscle and adipose tissues remains unclear. Here we show that *Chchd10* expression increases as myoblasts and preadipocytes differentiate. During myogenesis, CHCHD10 interacts with TAR DNA binding protein 43 (TDP-43) in regenerating myofibers in vivo and in newly differentiated myotubes ex vivo. Surprisingly, *Chchd10*^*KO*^ mice had normal skeletal muscle development, growth and regeneration, with moderate defects in grip strength and motor performance. *Chchd10*^*KO*^ similarly had no effects on development of brown and white adipose tissues (WAT). However, *Chchd10*^*KO*^ mice had blunted response to acute cold and attenuated cold-induced browning of WAT, with markedly reduced UCP1 levels. Together, these results demonstrate that *Chchd10* is dispensable for normal myogenesis and adipogenesis but is required for normal motility and cold-induced, mitochondrion-dependent browning of adipocytes. The data also suggest that human *CHCHD10* mutations cause myopathy through a gain-of-function mechanism.

**Supplementary Information:**

The online version contains supplementary material available at 10.1186/s13619-022-00111-0.

## Background

Skeletal muscle and adipose tissue (fat) are key peripheral metabolic organs that mediate glucose and fatty acid disposal (Cohen and Spiegelman 2016). They together comprise the largest endocrine system in the body, accounting for 50–80% of body weight, and secrete many myokines and adipokines into circulation to regulate systemic physiology (Coelho et al. 2013). These tissues also possess several other functions, as skeletal muscles are essential for mobility (Fujita et al. 2018) and adipose tissues are critical for thermal and mechanical insulation (Trayhurn 1993). For these reasons, dysfunction of muscle and fat often result in the pathogenesis of muscle wasting/weakness, obesity, insulin resistance and Type II diabetes (T2D) (Guilherme et al. 2008; Zierath et al. 2000). These conditions and associated metabolic syndromes, including cardiovascular diseases and hypertension, collectively affect quality of life for a large fraction (> 25%) of the world population (Bornfeldt and Tabas 2011; Zhou et al. 2017). Hence, understanding the cellular and molecular regulation of normal muscle and adipose tissue function is crucial for improving metabolic health of the affected population.

Mitochondria play a pivotal role in bioenergetics and metabolism in all cell and tissue types (Schrepfer and Scorrano 2016). Dysfunctional mitochondria with deficient ATP production in skeletal muscle and adipose tissue are associated with diseases (Boengler et al. 2017). Energy usage in skeletal muscles can aggrandize by over one hundred-fold instantly. In order to satisfy this energy requirement, cells of muscle tissue contain plentiful mitochondria, and transform nutrients into ATP (Russell et al. 2014). The skeletal muscle alone accounts for about 45% of body mass, representing a main site for disposal of glucose and fatty acids (Schenk and Horowitz 2007). Skeletal muscle can utilize both glycolysis and OXPHOS for the production of ATP, with preference of substrate utilization being determined by the mitochondrial density and the type of muscle fiber (i.e. glycolytic or oxidative myofibers) (Kunz 2001). Dysfunction of mitochondria were found in human skeletal muscles in type II diabetes (Kelley et al. 2002).

Based on mitochondria content and lipid droplet morphology and abundance, adipocytes can be classified as brown, beige or white adipocytes. While white adipocytes are the main storage site for energy in form of triglycerides (Rosen and Spiegelman 2014), brown adipocytes mainly function in the dissipation of energy as heat through UCP1-mediated thermogenesis (Roesler and Kazak 2020). Recently, UCP1-positive beige adipocytes with thermogenic potential were discovered in WAT in response to various stimuli, including cold treatment (Vitali et al. 2012; Wu et al. 2012). UCP1-positive adipocytes exist in metabolically healthy people, but patients with obesity usually lack these cells (Van et al. 2009; Saito et al. 2009). In this regard, conversion of white adipocytes to brown or beige adipocytes, namely browning or beiging, represents a potential therapy to treat metabolic illness.

Although the number of mitochondria is significantly lower in white adipocytes than in skeletal myocytes, mitochondrial function remains essential for adipocytes. Adipocyte differentiation is accompanied by elevated mitochondrial biogenesis and content (Wilson-Fritch et al. 2003). Moreover, obesity and T2D are often associated with abnormal mitochondrial function and decreased activity of the respiratory chain (Ritov et al. 2010; Kelley et al. 2002). Decreased expression of genes critical for mitochondrial oxidation metabolism were found in diabetic patients and non-diabetic patients with a family history of diabetes (Mootha et al. 2003; Patti et al. 2003). Furthermore, the ATP synthesis rate is decreased by 27%, and mitochondrial function is reduced by 45% in diabetic patients (Phielix et al. 2008). However, whether impaired mitochondrial function is a driver or consequence of diabetes is unclear.

CHCHD10 is a mitochondrial inner membrane protein thought to be crucial for mitochondrial cristae junctions (Koehler and Tienson 2009; Hell 2008), which are orchestrated by the cristae organizing system (MICOS) interacting with the SAM complex. The predicted biological functions of CHCHD10 include mitochondrial cristae formation (Genin et al. 2016a), protein transport (Imai et al. 2019), ATP synthesis (Martherus et al. 2010) and even reprogramming of induced pluripotent cells (Harjuhaahto et al. 2020). Importantly, mutations of the *CHCHD10* gene have been associated with frontotemporal dementia and/or amyotrophic lateral sclerosis-2 (FTD/ALS2), myopathy and spinal muscular atrophy (SMA) (Bannwarth et al. 2014a; Johnson et al. 2014). However, the function of this mitochondrial protein in development and function of muscle and adipose tissues is not well understood. In this study, we surveyed the expression patterns of *Chchd10* during myogenesis and adipogenesis, and generated *Chchd10* KO mice through CRISPR-CAS9 mediated gene targeting to investigate the in vivo role of CHCHD10. The evidence obtained in present study indicates that *Chchd10* is dispensable for myogenesis and adipogenesis, but essential for cold-induced browning of white adipose tissue in mice.

## Results

### Chchd10 expression in skeletal muscles and during myogenic differentiation

**Fig. 1:**
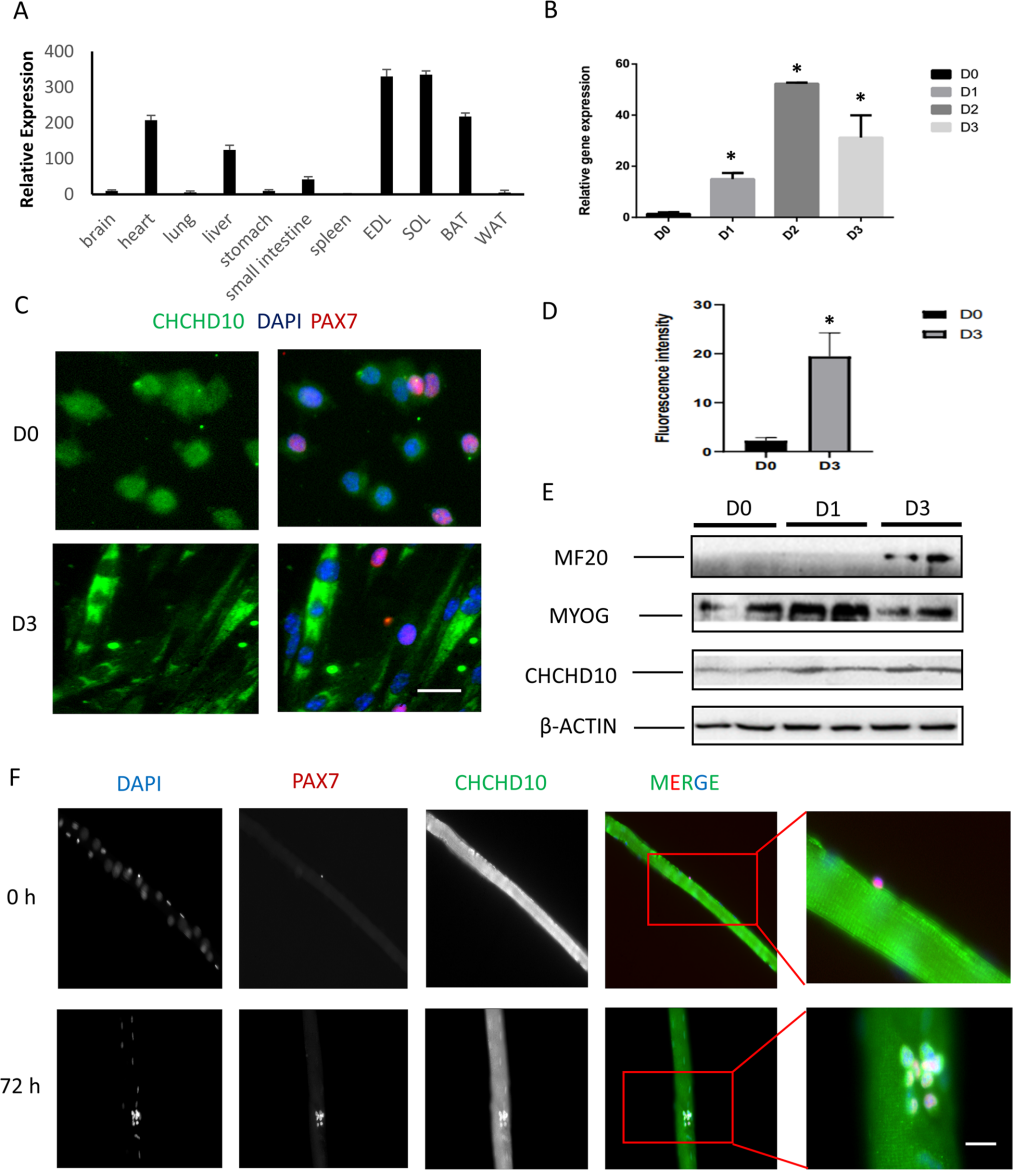
Chchd10 expression is enriched in skeletal muscles and upregulated during differentiation of satellite cell-derived primary myoblasts. **A** Relative mRNA levels of Chchd10 in various mouse tissue determined by qPCR, normalized to the mRNA level of the brain. *N* = 3. **B** Relative levels of Chchd10 mRNA in primary myoblasts at various days (D0-D3) after induced to differentiate. * *p* < 0.05 compared to D0 value. *N* = 3. **C** Immune staining of CHCHD10 (green) and PAX7 (red) in myoblasts before (D0) and after (D3) differentiation, scale bar: 20 μm. **D** Fluorescence intensity of CHCHD10 in myoblasts before (D0) and after (D3) differentiation. **E** Protein level of MF20, MyoG, CHCHD10 and β-ACTIN in D0, D1 and D3 differentiating myoblasts. **F** Immune staining of CHCHD10 (green) and PAX7 (red) in quiescent (0 h) and activated (72 h) satellite cells cultured on myofibers, scale bar: 20 μm. Nuclei were labeled with DAPI. The cluster of cells at 72 h contains self-renewed, proliferating and differentiated cells

We examined the relative mRNA levels of *Chchd10* using quantitative PCR (qPCR) to reflect *Chchd10* gene expression in different tissues from 8-week-old mice. High levels of *Chchd10* were found in mitochondria enriched tissues, including skeletal muscle, brown adipose tissue, liver and heart (Fig. 1A). To determine the dynamics of *Chchd10* expression during myogenic differentiation, we isolated primary myoblasts from adult mice and examined *Chchd10* mRNA levels in myoblasts during proliferation (D0) and differentiation (D1–3) (Fig. 1B). *Chchd10* levels increased during myogenic differentiation, peaked at D2 with a level that is about 50 times of that in proliferating myoblasts (Fig. 1B). Immunofluorescence staining further confirmed that CHCHD10 immunofluorescence is very low in PAX7^+^ myoblasts but very high in newly differentiated myotubes (Fig. 1C). The immunofluorescence intensity of PAX7^+^ myoblasts and differentiated myotubes were quantified in Fig. 1D. Further, western blot analysis showed protein level of CHCHD10 was also increased when myoblasts differentiation proceeds (Fig. 1E). These results indicate that *Chchd10* is dynamically expressed during myogenic differentiation.

We also examined CHCHD10 expression in quiescent and activated satellite cells (SCs) attached to single myofibers isolated from the extensor digitorum longus (EDL) muscles of adult mice. *Chchd10* immunofluorescence was not detectable in PAX7 positive quiescent SCs located on freshly isolated EDL myofibers that were immediately fixed after isolation (Day 0 in Fig. 1F). We subsequently cultured the EDL myofibers in suspension for 3 days, during which SCs activate, proliferate and differentiate. CHCHD10 immunofluorescence was detected in clusters of SC progenies, in both PAX7^+^ and PAX7^−^ cells (Fig. 1F). These results confirm that CHCHD10 is not expressed in quiescent SCs but exhibits elevated expression during activation and differentiation of SCs.

### CHCHD10 interacts with TAR DNA binding protein 43 (TDP-43) in nascent muscle cells

**Fig. 2:**
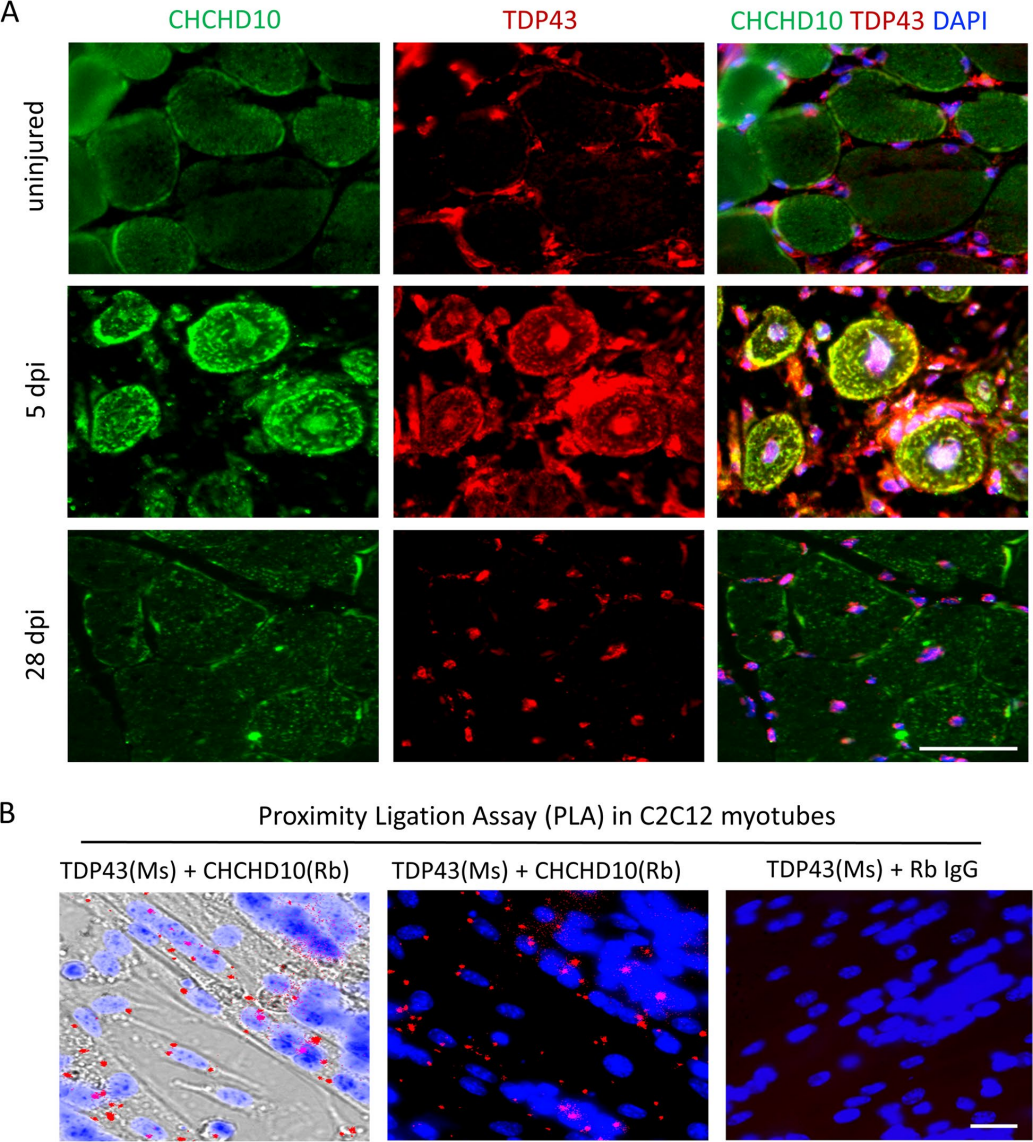
CHCHD10 is highly expressed in nascent myotubes where it interacts with TDP43. **A** Immunofluorescence of CHCHD10 and TDP43 in TA muscles at 0 (uninjured), 5 and 28 day post injury (dpi), scale bar: 100 μm. **B** Proximity ligation assay in C2C12 myotubes using rabbit anti-CHCHD10 and mouse anti-TDP43 antibodies, scale bar: 20 μm

*CHCHD10* mutations associated FTD/ALS pathology are characterized by TDP-43 granules (Woo et al. 2017), we therefore sought to determine if CHCHD10 interacts with TDP-43 in muscle cells. We first performed co-immunofluorescent labeling of CHCHD10 with TDP-43 in non-injured, nascent and regenerated myofibers (Fig. 2A). In uninjured adult TA muscle, TDP-43 immunofluorescence was mainly found in myonuclei and nuclei of interstitial cells, while weak CHCHD10 signal was only detected in diffusive form in the myofiber cytoplasm (Fig. 2A, top row). In newly regenerated nascent myofibers at 5 days post-injury (dpi), however, intense CHCHD10 signals were detected in granular form in the myofiber cytoplasm and in the myonuclear envelope, co-localized perfectly with newly emerged myofiber cytoplasmic TDP43 granules (Fig. 2A, middle row). The interstitial TDP43 signal remained similar to the pre-injury level (Fig. 2A). At 28 dpi, when muscle regeneration is completed and muscle function is recovered (Fig. 1A, bottom row), the distribution of TDP-43 and CHCHD10 resembled the patterns found in uninjured muscles. These results reveal previously unappreciated transient CHCHD10 granules that co-localize to TDP43 puncta, also known to be transiently formed in nascent myofibers during regeneration (Vogler et al. 2018).

To further investigate if CHCHD10 and TDP-43 interact with each other, we performed a proximity ligation assay (PLA) in newly formed C2C12 myotubes. An interaction would be manifested by a punctum in this assay. The PLA revealed numerous CHCHD10–TDP-43 interaction puncta, located in the cytoplasm of myotubes (Fig. 2B, left and middle panels). No interaction puncta were detected in the negative control group (Fig. 1B, right) or in mononuclear cells (non-myotubes) (Fig. 2B left). Therefore, there is a temporal and spatial association between CHCHD10 and TDP-43 in nascent muscle cells.

### Deletion of Chchd10 has no effect on muscle development or regeneration

**Fig. 3:**
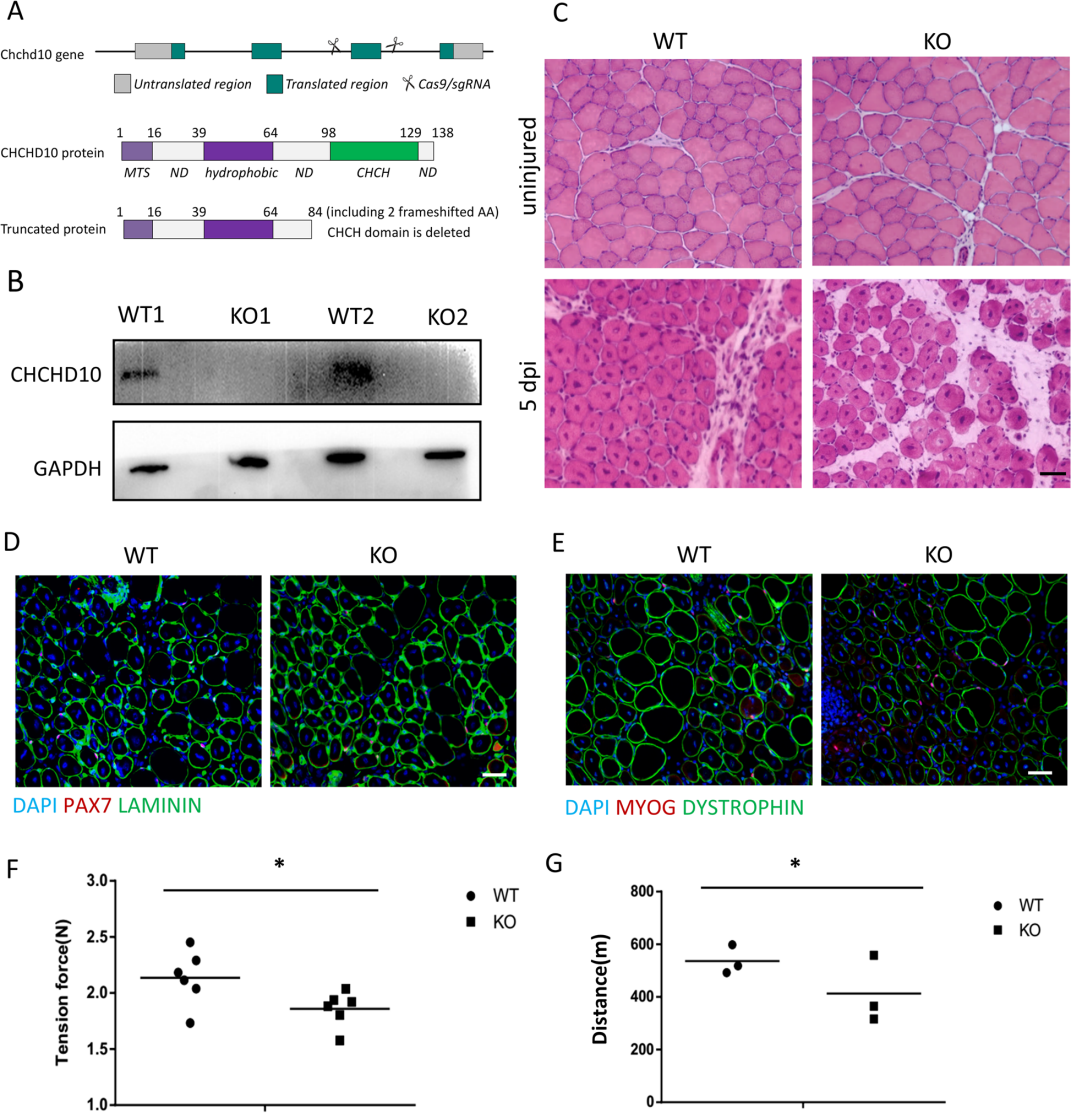
*Chchd10* knockout does not affect muscle development and satellite cell mediated muscle growth and regeneration; but reduces grip strength and exercise performance in mice. **A** *Chchd10* knockout (KO) strategy using CRISPR-CAS9 targeting to delete Exon 3 which truncated the functional CHCH domain. MTS: mitochondrial targeting sequence; ND: non-domain sequence. **B** Western blot confirming the lack of CHCHD10 protein in the KO TA muscles. *N* = 3. **C** H&E cross-sectional staining of representative regenerated areas in uninjured (upper) and injured TA muscles at 5 day post injured (dpi; bottom) TA muscle, scale bar: 100 μm. **D-E** Double immunofluorescence staining of PAX7 and α-LAMININ (**D**, to mark satellite cells), or MYOG and DYSTROPHIN (**E**, to mark newly differentiated myoblasts) in TA muscle cross-sections at 5 dpi. Scale bar: 100 μm. **F-G** Grip strength (**F**) and treadmill running distance (**G**) for WT and *Chchd10*^*KO*^ mice. * *p* < 0.05. *N* = 6 for **F** and *n* = 3 for **G**

To investigate the function of CHCHD10 in vivo, we employed CRISPR-CAS9 to remove exon 3 of the *Chchd10* gene, which is predicted to result in a truncated peptide without the functional CHCH domain (Fig. 3A). The KO mice were born healthy at the expected Mendelian ratio (25%) from heterozygous breeding pairs. Postnatal growth of the KO mice was also normal, as manifested by similar body weight and body composition between KO and WT littermate controls (data not shown). We performed western blots to confirm the absence of the CHCHD10 protein in KO muscles (Fig. 3B). We then performed histological analyses of TA muscles by H&E staining, showing similar myofiber size, fiber type, and fiber number per muscle between WT and KO mice (Fig. 3C), indicating that CHCHD10 is dispensable for muscle development and growth.

To determine whether *Chchd10* deficiency impairs muscle regeneration, we injured TA muscles in adult WT and *Chchd10*^KO^ mice with cardiotoxin (CTX), a standard protocol to induce muscle regeneration. H&E and immunostaining with antibodies against LAMININ (for myofiber extracellular matrix), DYSTROPHIN (for myofiber membrane) PAX7 (for SCs) and MYOG (for differentiated myonuclei) showed no difference between WT and KO muscles at 5 and 28 dpi (Fig. 3C-E). Specifically, although dystrophin signal appears weaker in KO TA muscles (Fig. 3E), both WT and KO TA muscles contained similar numbers and sizes of regenerated myofibers (indicated by central nuclei) and a similar abundance of PAX7^+^ and MYOG^+^ cells at 5 dpi (Fig. 3D–E). These results demonstrate that CHCDH10 is dispensable for postnatal muscle regeneration.

We also performed grip strength and treadmill running tests on WT and *Chchd10*^KO^ mice. Both grip strength and running distance were significantly lower in the *Chchd10*^KO^ mice compared to their WT littermates (Fig. 3F, G). As *Chchd10* knockout had no effect on muscle development and regeneration, these results suggest that CHCHD10 may be involved in regulating the contractile function and motor performance of skeletal muscles through motor neurons.

### Chchd10 expression pattern during adipogenic differentiation

**Fig. 4:**
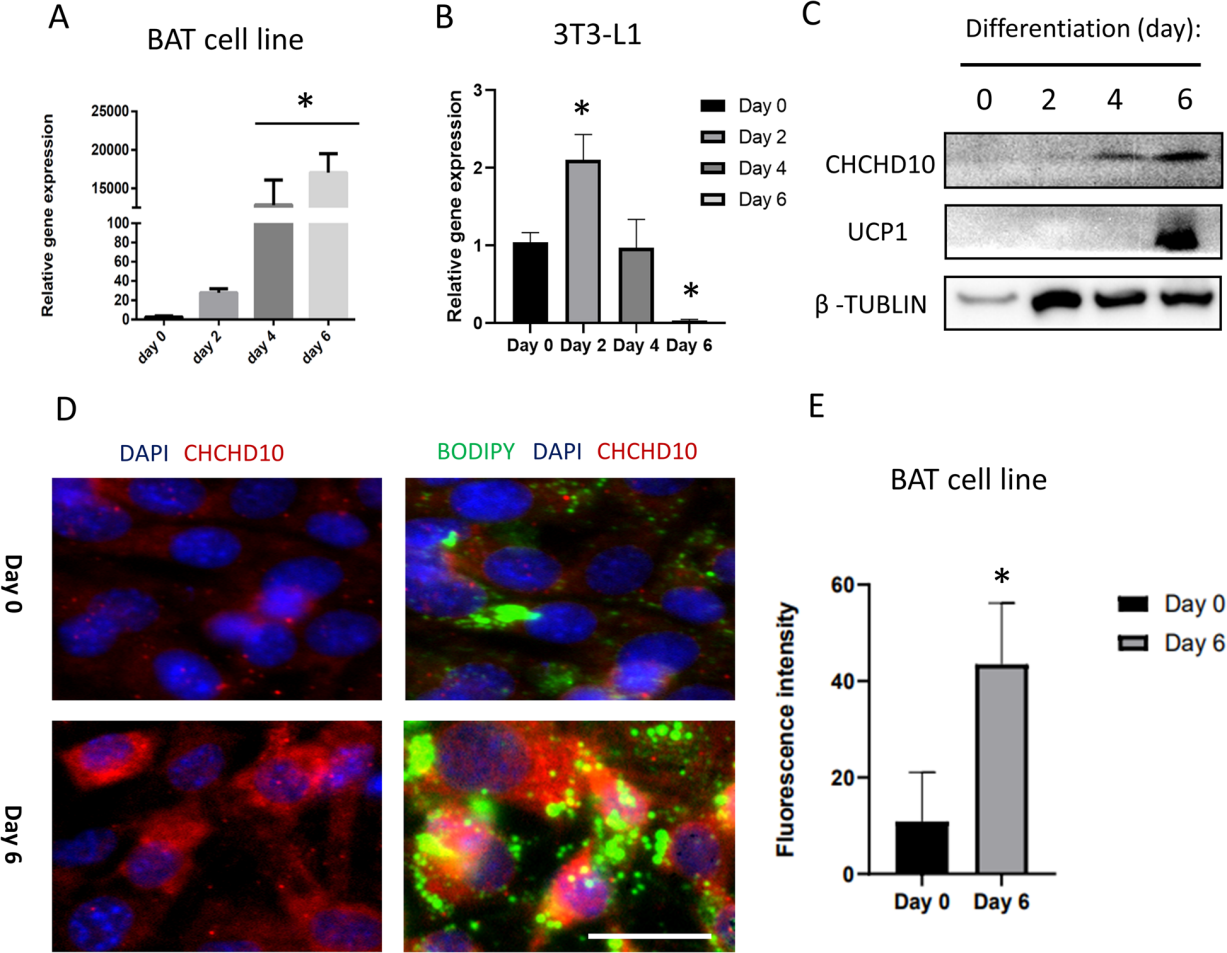
Expression of *Chchd10* during adipogenic differentiation. **A-B** Expression of *Chchd10* at various days during differentiation of a murine brown preadipocyte cell line (**A**) and a murine white preadipocyte cell line (3T3-L1) (**B**). * *p* < 0.05, *n* = 3. **C** Protein level of CHCHD10, UCP1 and β-TUBLIN in D0, D2, D4 and D6 differentiating brown adipose SVF cells. **D** Immunostaining of CHCHD10 (Red) and Bodipy (Green) in undifferentiated (Day 0) and differentiated (Day 6) brown adipose tissue cell line, scale bar: 20 μm. Nuclei were counterstained with DAPI in blue. **E** Fluorescence intensity of CHCHD10 in undifferentiated (Day 0) and differentiated (Day 6) brown adipose tissue cell line

The high expression of *Chchd10* in BAT and low expression of *Chchd10* in WAT (Fig. 1A) suggest that CHCHD10 may function differently in brown and white adipose tissues. To address this possibility, we examined *Chchd10* expression during adipogenic differentiation of a BAT cell line. There was minimal expression of *Chchd10* in BAT preadipocytes at Day 0, before induction of differentiation (Fig. 4A). However, *Chchd10* levels increased rapidly after induction of differentiation, reaching a level over 15,000-fold higher at Day 6 compared to Day 0 (Fig. 4A). We also examined *Chchd10* expression during differentiation of the 3T3-L1 white preadipocyte cell line. The level of *Chchd10* transiently increased by 2-fold at Day 2 of differentiation, followed by a decrease in *Chchd10* expression, resulting in a more than 90% reduction in *Chchd10* expression at Day 6 compared to Day 0 (Fig. 4B). Western blot analysis showed protein level of CHCHD10 was also increased when BAT SVF cells differentiation proceeds (Fig. 4C). We further used immunofluorescence staining to examine the level of CHCHD10 in undifferentiated and differentiated murine brown preadipocytes. CHCHD10 immunofluorescence signal was barely detectable in undifferentiated preadipocytes, but was very strong in differentiated adipocytes containing lipid droplets (Fig. 4D). The immunofluorescence intensity of CHCHD10 in undifferentiated and differentiated BAT cell line were quantified in Fig. 4E. These results indicate a potential role of CHCHD10 in brown adipocytes.

### Chchd10 mutation had no effect on development of adipose tissues

**Fig. 5:**
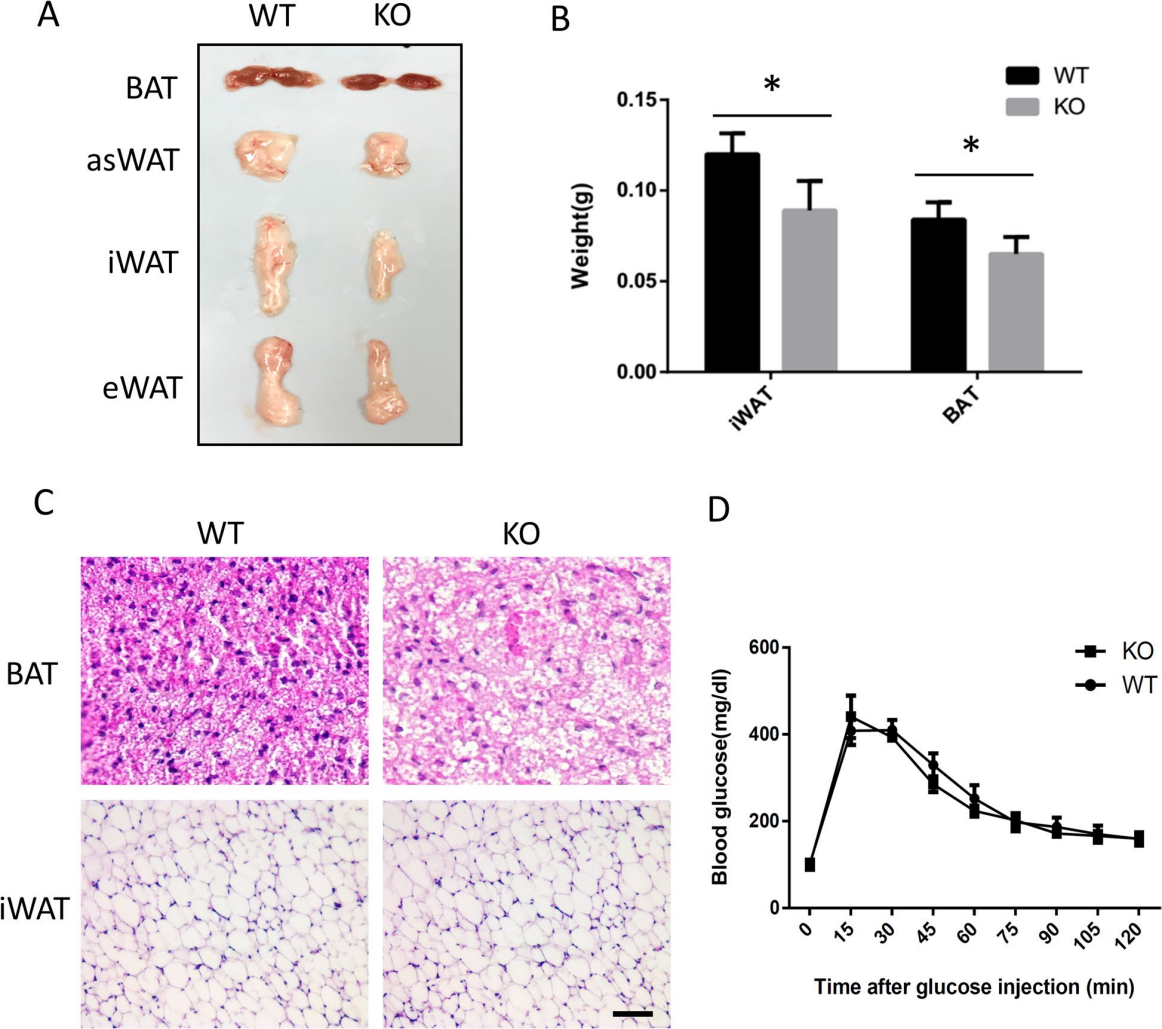
*Chchd10* KO reduces expansion of adipose tissues but does not affect systemic glucose homeostasis. **A** Representative images of intrascapular brown adipose tissue (BAT), anterior subcutaneous white adipose tissue (asWAT), inguinal WAT (iWAT) and epididymal WAT (eWAT) isolated from wild type and *Chchd10*^*KO*^ littermate mice at 3-month-old age. **B** Average weight of BAT and iWAT in wild type and *Chchd10*^*KO*^ mice. *N* = 3. **C** Representative areas of H&E staining of BAT and iWAT cross-sections in wild type and *Chchd10*^*KO*^ mice. Scale bar: 100 μm. **D** Glucose Tolerance test (GTT) for wild type and *Chchd10*^*KO*^ mice after interperitoneal injection of 1 g/kg glucose. Animals were fasted overnight before GTT

To understand the potential role of CHCHD10 in adipose tissue development and expansion, we examined various fat depots in *Chchd10* KO and WT mice at several ages. All fat depots are developed and formed normally in young mice (data not shown). At 3 months old, the BAT, asWAT (anterior subcutaneous white adipose tissue lateral to the BAT), iWAT (inguinal WAT) and eWAT (epididymal WAT) all appeared smaller (Fig. 5A), indicative of defective fat expansion. The weight of iWAT and BAT were significantly lower in *Chchd10*^*KO*^ mice than in WT mice (Fig. 5B). Histologic analyses of BAT and iWAT were further performed, showing an obvious enlargement of lipid droplets in the *Chchd10*^*KO*^ BAT compared to WT BAT (Fig. 5C, top). However, no obvious difference can be found in adipocyte size and morphology of iWAT between WT and *Chchd10*^*KO*^ mice (Fig. 5C, bottom).

We also examined if the reduced adipose tissue mass and slight increase in lipid droplets in BAT affect systemic glucose homeostasis by performing a glucose tolerance test (GTT). Compared to their WT littermates, *Chchd10*^*KO*^ mice had similar blood glucose levels after injection of glucose (Fig. 5D). Therefore, the moderate reduction in the size of fat depots in *Chchd10* KO mice did not significantly affect glucose homeostasis in the adult mice.

### *Chchd10*^*KO*^ mice have impaired cold-induced browning of iWAT and acute cold responses

**Fig. 6:**
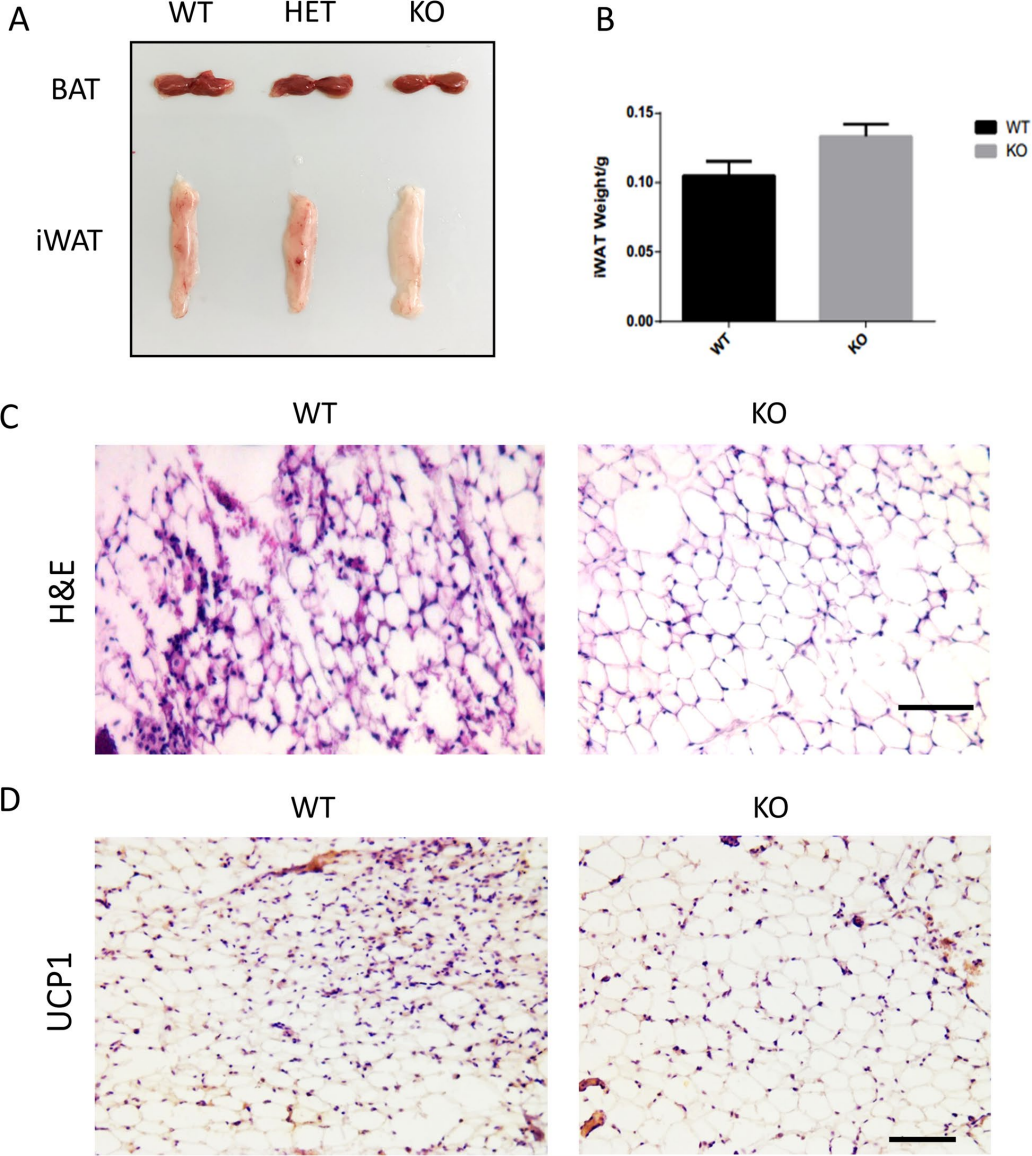
*Chchd10* KO attenuates cold-induced browning of subcutaneous inguinal white adipose tissues (iWAT). **A** Representative images of BAT and iWAT in WT and *Chchd10*^*KO*^ mice after 7 days of cold treatment at 6 °C. **B** Average weights of iWAT in wildtype and *Chchd10*^*KO*^ mice after cold treatment. *N* = 4. **C** Representative H&E staining images of iWAT cross-sections in WT and *Chchd10*^*KO*^ mice after cold treatment (scale bar: 100 μm). **D** Representative UCP1 staining of iWAT cross-sections in WT and *Chchd10*^*KO*^ mice after cold treatment (scale bar: 100 μm)

We further examined if *Chchd10* knockout affects cold-induced browning of WAT by subjecting WT and *Chchd10*^*KO*^ mice to cold treatment at 6 °C in an environmental chamber for 7 days. No obvious differences were observed in the gross morphology of BAT among WT, heterozygous (HET) and KO mice after cold treatment (Fig. 6A). However, the *Chchd10*^*KO*^ iWAT appeared slightly larger (without reaching a significant difference in weight) and less brown compared to WT and HET iWAT (Fig. 6A–B). H&E staining revealed cold-induced formation of small and multilocular beige adipocytes in the iWAT of WT mice but not in *Chchd10*^*KO*^ iWAT (Fig. 6C). In addition, immunohistochemistry (IHC) staining of UCP1 revealed a relatively higher UCP1 signal intensity in WT iWAT than in *Chchd10*^*KO*^ iWAT (Fig. 6D). These results demonstrate that *Chchd10* mutation attenuates cold-induced browning of iWAT in vivo.

**Fig. 7:**
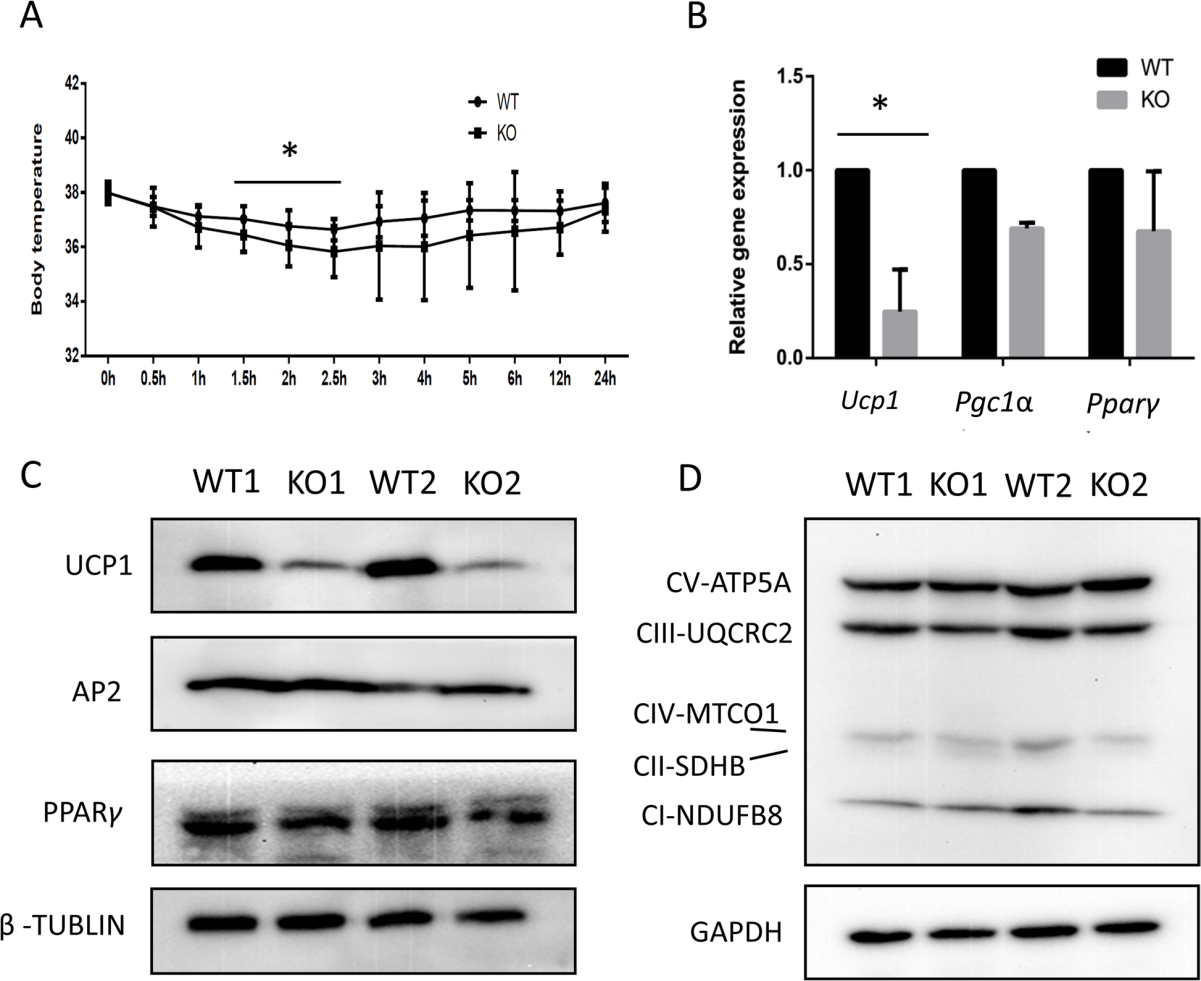
*Chchd10* KO mice had impaired cold tolerance and reduced level of UCP1 in subcutaneous iWAT after cold treatment. **A** Rectal core temperature of WT and *Chchd10*^*KO*^ mice in the first 24 h after challenged by cold exposure (6 °C). Food was provided during cold-exposure. * *p* < 0.05, *n* = 6. **B** Quantitative PCR detection of the relative mRNA levels of *Ucp1, Pgc1*α and *Pparγ* in iWAT of WT and *Chchd10*^*KO*^ mice after 7 days of cold treatment. * *p* < 0.05, *n* = 3. **C** Western blot showing relative protein levels of UCP1, AP2, PPAR*γ* in iWAT of WT and *Chchd10*^*KO*^ mice after cold treatment. **D** Western blot showing relative abundance of mitochondrial Complex I-V proteins in iWAT of WT and *Chchd10*^*KO*^ mice after cold treatment

We also examined the acute cold response of WT and *Chchd10*^*KO*^ mice when exposed to 6 °C for 24 h. The results showed a small but significant decrease in the rectal temperature between 1.5 and 2.5 h of cold exposure in the *Chchd10*^*KO*^ mice (Fig. 7A). The body temperatures of *Chchd10*^*KO*^ mice eventually reached that of WT mice within 24 h of cold exposure (Fig. 7A). To understand the molecular basis of the reduced cold response, we examined the mRNA levels of *Ucp1, Pgc1α* and *Pparγ*, genes involved in thermogenesis, mitochondrial biogenesis and adipogenesis. The level of *Ucp1* in iWAT was about 75% lower in *Chchd10*^*KO*^ mice compared to the WT mice, but the levels of *Pparγ* and *Pgc1α* were not significantly different for WT and *Chchd10*^*KO*^ iWAT after cold exposure (Fig. 7B). Western blot analysis also showed that UCP1 expression was lower in *Chchd10*^*KO*^ mice than in WT mice, with no significant difference in the levels of AP2 (FABP4) and PPARγ (Fig. 7C). Consistent with similar levels of *Pgc1α* in WT and *Chchd10*^*KO*^ mice, there were no obvious differences in the expression of mitochondrial electron transport chain complex (CI–CV) proteins (Fig. 7D). Taken together, these results indicate that *Chchd10* mutation attenuates cold-induced browning of iWAT and impairs the acute cold response of *Chchd10*^*KO*^ mice due to reduced UCP1 levels.

## Discussion

Previous studies have shed light on the function of CHCHD10 in other cell and tissue types. *CHCHD10* mutations lead to MICOS complex disassembly and loss of mitochondrial cristae with a decrease in nucleoid number and nucleoid disorganization (Genin et al. 2018). CHCHD10 KO fibroblasts have impaired mitochondrial genome maintenance after oxidative stress, which may explain why abnormal mtDNA molecules accumulate in *CHCHD10* mutant patient muscles (Brockmann et al. 2018; Genin et al. 2016b; Straub et al. 2018). CHCHD10 was also found to accumulate with its paralog CHCHD2 in aggregates in brain tissues of CHCHD10^S55L^ mice. These aggregates induce a potent mitochondrial integrated stress response (mtISR) through mTORC1 activation (Anderson et al. 2019). However, CHCHD10 ablation fails to induce mtISR and does not lead to any disease pathology, indicating that CHCHD10^S55L^-dependent disease pathology is due to a gain-of-function mechanism (Anderson et al. 2019; Ronchi et al. 2015). The lack of an overt developmental phenotype in *Chchd10*^*KO*^ mice may be due to compensation by CHCHD2, a closely related paralog, as recent reports have shown similar nuclear localization and transcriptional function of CHCHD10 and CHCHD2 (Woo et al. 2017; Aras et al. 2015; Aras et al. 2017). The presence and function of CHCHD10 and CHCHD2 seem to be actively coordinated in cells (Burstein et al. 2018). Antibody used in present study recognizes AA82–142 epitope (CHCH domain) of this protein. As shown in our knockout strategy (Fig. 3A), the CHCH domain is entirely ablated in the knockout mice. Alternatively, the remaining 1–84 AA truncated peptide may be functionally sufficient for the role of full-length CHCHD10 in muscle cells, leading to the lack of apparent phenotype.

The grip strength and treadmill running distance of *Chchd10*^*KO*^ mice were significantly reduced. Since we used global *Chchd10*^*KO*^ mice, we cannot distinguish if the defect in motor performance is due to skeletal muscle contractile defects or motoneuron defects. As we did not observe any defects in muscle mass, morphology and gene expression, we speculate that the reduced exercise performance is mainly due to motoneuron dysfunction, especially considering that CHCHD10 is critical for motor neuron function (Penttilä et al. 2015). Similar to our results, reduced grip strength was also discovered in patients with a *Chchd10* mutation (Penttilä et al. 2015). Motor defects and abnormal neuromuscular transmission were reported in a conditional knockout of *Chchd10* mice, consistent with the notion that mitochondrial CHCHD10 is required for ATP production (Xiao et al. 2019). A potential function of CHCHD10 at synapses and neuromuscular junctions could also explain the defective motor performance in the *Chchd10*^*KO*^ mice in present study.

In comparison to WTs, white adipocytes of *Chchd10*^*KO*^ mice showed reduced *Ucp1* expression after cold treatment. White adipocyte browning induced by cold stress correlates with the formation of UCP1 positive adipocytes, which are thermogenic and have several features similar to brown adipocytes (Schulz et al. 2010). In our result, reduced *Ucp1* expression in *Chchd10*^*KO*^ mice under cold stress suggests a role for CHCHD10 in thermogenesis. Adipocyte mitochondria are crucial for cold-induced browning, where fatty acid oxidation and thermogenesis take place (Peirce et al. 2014). CHCHD10 is found at both the mitochondrial inner membrane and within mitochondrial cristae (Koehler and Tienson 2009; Hell 2008), and its mutations result in loss of cristae junctions (Bannwarth et al. 2014b). Deletion of *Chchd10* reduces mitochondria function, without affecting levels of OXPHOS proteins in our study (Fig. 7D), suggesting that CHCHD10 participates in mitochondria function to affect thermogenesis.

To promote thermogenesis, UCP1 dissipates the mitochondrial proton motive power produced by the respiratory chain (Kazak et al. 2017). A human *CHCHD10* mutation resulted in ultrastructural mitochondrial aberrancy, damaged respiratory chain function and alteration of mitochondrial DNA (Bannwarth et al. 2014b). Taken together, the *Chchd10* mutation would most likely also affect the mitochondrial respiratory chain activity, associated with ultrastructural mitochondrial abnormalities, lower UCP1 expression and blunted activation under cold stress. The global *Chchd10* KO may also affect the function of the sympathetic nervous system that is crucial for regulating thermogenesis. Therefore, adipose tissue specific conditional knockout mouse models are warranted to address the specific role of CHCHD10 in brown fats in future studies.

## Conclusions

In the present study, we used a *Chchd10* global KO mouse model to investigate the function of CHCHD10 in skeletal muscle and adipose tissues. We found that, while *Chchd10* KO did not affect the development and injury-induced regeneration of skeletal muscles, it did affect the motor performance of mice. Additionally, while various fat depots developed normally, the postnatal expansion and cold-induced browning of iWAT was attenuated in *Chchd10*^*KO*^ mice, resulting in their impaired ability to maintain body temperature in response to acute cold exposure. These results demonstrate multiple roles of CHCHD10 in adipocyte and skeletal muscle cell functions that are not due to defects in tissue differentiation.

Since CHCHD10 has a very short half-life time and degrades within hours (Burstein et al. 2018; Huang et al. 2018), it is unlikely a structural protein. Previous work has indicated that CHCHD10 most likely functions as a chaperone for protein import. CHCHD10 is implicated in neurodegenerative diseases as inducing aggregation of TDP-43 (McCann et al. 2020), a pathological feature of several neurodegenerative diseases (Woo et al. 2017; Neumann et al. 2007) Moreover, amyloid-like myo-granules were formed by TDP-43 and RNA in regenerating muscle (Vogler et al. 2018). Our results also show that CHCHD10 interacts with TDP-43 in muscle cells, suggesting the possibility that CHCHD10 may transport TDP-43 into muscle cell nuclei. Interestingly, ablation of TDP-43 downregulates Tbc1d1 and alters adipose tissue metabolism (Chiang et al. 2010). Another study showed that TDP-43 can regulate fat deposition and glucose homeostasis (Stallings et al. 2013). Therefore, it is possible that *Chchd10* mutation disrupts TDP-43 expression or location in adipocytes, leading to the abnormal browning of iWAT.

## Methods

### Animal

*Chchd10* mutant mice (*Chchd10*^*KO*^) were generated using CRISPR–CAS9 gene editing strategy. All interventions and animal care procedures were performed in accordance with the Guidelines and Policies for Animal Surgery provided by Purdue University (West Lafayette, USA). Protocols were approved by the Institutional Animal Use and Care Committee. Mice were housed in the animal facility at room temperature (24–25 °C) with free access to water and standard rodent chow. Adult mice (2–3 months old) of both genders were randomly selected for experiments.

### Stromal vascular fraction (SVF) and 3T3-L1 cell culture

Primary inguinal white adipose (iWAT) SVF cells were isolated using collagenase digestion, followed by density separation. Briefly, the iWAT was minced and digested in 1.5 mg/ml collagenase at 37 °C for 30 min and 1 h, respectively. The digestions were terminated with DMEM containing 10% FBS and filtered through 100-μm filters to remove connective tissues and undigested trunks of tissues. Cells were then centrifuged at 450 g for 5 min to separate the SVF cells in the sediment and lipid-containing adipocytes in the floating layer. The freshly isolated SVF cells were seeded and cultured in growth medium containing DMEM, 20% FBS and 1% penicillin/streptomycin (P/S) at 37 °C with 5% CO_2_ for 3 days, followed by feeding with fresh medium every 2 days. 3T3-L1 cells were cultured in DMEM with 10% FBS. For SVF cell adipogenic differentiation, the cells were induced with induction medium containing DMEM, 10% FBS, 2.85 μM insulin, 0.3 μM dexamethasone, 1 μM rosiglitazone, and 0.63 mM 3-isobutyl-methylxanthine for 3 days and then treated with differentiation medium containing DMEM, 10% FBS, 200 nM insulin and 10 nM T3 for 4 days until adipocytes matured. To avoid the effect of cell density on adipogenic differentiation, cells were induced to differentiate when they reach 90% confluency. For 3T3-L1 adipogenic differentiation, cells with 100% confluency were kept in growth medium for 2 days and treated with induction medium for 2 days, followed by differentiation medium (without T3) for 6 days. Mycoplasma was certified when cells were purchased. All cell lines were periodically tested for identity using PCR and by appearance of morphological features.

### Primary myoblast culture and differentiation

Primary myoblasts were isolated from hind limb skeletal muscles of wild type 4–6-week-old mice. Muscles were minced and digested in type I collagenase and Dispase B mixture (Roche Applied Science). The digestions were stopped with F-10 Ham’s medium containing 20% FBS. Cells were then filtered from debris, centrifuged and cultured in growth medium (F-10 Ham’s medium supplemented with 20% FBS, 4 ng/ml basic fibroblast growth factor, and 1% penicillin–streptomycin) on collagen-coated cell culture plates at 37 °C, 5% CO_2_. For differentiation, primary myoblasts were seeded on BD Matrigel-coated cell culture plates and induced to differentiate in differentiation medium (DMEM supplemented with 2% horse serum and 1% penicillin-streptomycin).

### Single myofiber isolation and culture

Single myofibers were isolated from EDL muscles of adult mice (Pasut et al. 2013). In brief, EDL muscles were dissected carefully and subjected to digestion with collagenase I (2 mg/mL, Sigma) in Dulbecco’s Modified Eagle’s Medium (DMEM, Sigma) for 1 h at 37 °C. Digestion was stopped by carefully transferring EDL muscles to a pre-warmed Petri dish (60-mm) with 6 mL of DMEM, and single myofibers were released by gently flushing muscles with a large bore glass pipette. Released single myofibers were then transferred and cultured in a horse serum-coated Petri dish (60-mm) in DMEM supplemented with 20% fetal bovine serum (FBS, HyClone), 4 ng/mL basic fibroblast growth factor (Promega), and 1% penicillin-streptomycin (HyClone) at 37 °C for indicated days.

### Muscle injury and regeneration

Muscle regeneration was induced by injection of cardiotoxin (CTX; Millipore Sigma, Burlington, MA, USA) as previously described by Yue et al. (Yue et al. 2017) . Briefly, mice were anesthetized using a ketamine-xylazine cocktail, and then 50 μL of 10 μM CTX was injected into the left tibialis anterior (TA) muscle. Muscles were then harvested at indicated times post-injection to assess the completion of regeneration and repair.

### Paraffin and Cryosection and hematoxylin-eosin staining

Fresh TA, soleus (Sol), and EDL muscles were embedded in optimal cutting temperature compound and frozen in isopentane chilled on dry ice. Then, the tissues were cut at 10-μm thickness by Leica CM1850 cryostat (Leica Biosystems, Wetzlar, Germany). Adipose tissues were fixed in 10% formalin for 24 h at room temperature. Then, the adipose tissues were embedded in paraffin and cut into 4-μm thickness slices, deparaffinized, and rehydrated using xylene, ethanol, and water by standard methods. For hematoxylin and eosin (H&E) staining, the sections were stained in hematoxylin (30 min), rinsed in running water, and stained in eosin (1 min). H&E staining images were captured with a Nikon D90 digital camera (Nikon, Tokyo, Japan) mounted on a microscope.

### Immunostaining

Immunohistochemistry was performed on a Dako Autostainer (Agilent, Santa Clara, CA, USA). Slides were incubated with 3% hydrogen peroxide and 2.5% normal horse serum (S-2012;Vector Laboratories, Burlingame, CA, USA) followed by incubation with rabbit polyclonal anti-UCP1 primary antibody diluted 1:200 in 2.5% normal horse serum (S-2012; Vector Laboratories) for 60 min. Signals were detected with an anti-rabbit IgG Polymer Detection Kit (MP-7401; Vector Laboratories). Labeling was visualized with 3,3′-diaminobenzidine as the chromogen (SK-4105; Vector Laboratories). Slides were counterstained with Harris hematoxylin (EK Industries, Joliet, IL, USA), and whole-slide digital images were collected at × 20 magnification with an Aperio Scan Scope Slide Scanner (Leica Biosystems).

### Total RNA extraction and real-time PCR

Total RNA was extracted from tissues using Trizol reagent (Thermo Fisher Scientific, Waltham, MA, USA) according to the manufacturer’s instructions. Then, 2 μg total RNA was reverse transcribed using random primers with Moloney murine leukemia virus reverse transcriptase (Thermo Fisher Scientific). Realtime PCR was carried out in a CFX96 Touch Real-Time PCR Detection System (Bio-Rad, Hercules, CA, USA) with SYBR Green Master Mix (Takara Bio, Kyoto, Japan). The specific gene primer sequences were listed in Supplemental Table 1. The 2^-ΔΔCt^ method was used to analyze the relative changes in gene expression normalized against 18S ribosomal RNA expression.

### Protein extraction and western blot analysis

Total protein was isolated from cells using RIPA buffer containing 25 mM Tris-HCl (pH 8.0), 150 mM NaCl, 1 mM EDTA, 0.5% NP-40, 0.5% sodium deoxycholate and 0.1% SDS. Protein concentrations were determined using Pierce BCA Protein Assay Reagent (Pierce Biotechnology). Proteins were separated by SDS-PAGE, transferred to a polyvinylidene fluoride membrane (Millipore Corporation), blocked in 5% fat-free milk for 1 h at room temperature and then incubated with primary antibodies in 5% milk overnight at 4 °C. Membranes were then incubated with secondary antibody for 1 h at room temperature. Immunodetection was performed using enhanced chemiluminescence western blotting substrate (Santa Cruz Biotechnology) and detected with a FluorChem R System (Proteinsimple). Results shown in the figures are representative results from three independent experiments. Antibody information was listed in Supplementary Table 2.

### Blood glucose measurements

For GTT, mice were injected intraperitoneally with 100 mg/ml D-glucose (2 g/kg body weight) after overnight fasting, and tail blood glucose concentrations were measured by a glucometer (Accu-Check Active, Roche). Each mouse was randomly placed in a separate cage with a blinded ID number.

### Treadmill training

The treadmill practice was performed as described by Castro et al. (Castro and Kuang 2017) Mice were trained on the treadmill (Eco3/6 treadmill; Columbus Instruments, Columbus, OH, USA) with a fixed 10% slope at a constant speed of 18 m/min for 60 min daily, five days/week for 8 weeks before testing.

### Proximity ligation assay

For the proximity ligation assay, samples were incubated with indicated antibodies at the concentrations listed above. Secondary antibody incubation and Duolink proximity ligation assays were performed according to the manufacturer’s protocol (Sigma-Aldrich).

### Data analysis

For analysis of phenotypes, all comparisons were made between pairs of WT and KO littermates of the same gender (both genders were included). All data are presented as mean ± SEM. Statistical analyses were made by two-tailed Student’s t tests. Values of *P* < 0.05 were considered significant. All the experiments were repeated at least three times.

## Supplementary Information

**Additional file 1.**

## Data Availability

All data generated or analyzed during this study are included in this published article.

## Abbreviations

iWAT: Inguinal white adipose tissue
eWAT: Epididymal white adipose tissue
asWAT: Anterior subcutaneous white adipose tissue
BAT: Brown adipose tissue
TDP-43: TAR DNA binding protein 43
Chchd10: Coiled-coil-helix-coiled-coil-helix-domain containing 10
OXPHOS: Mitochondrial oxidative phosphorylation
IHC: Immunohistochemistry
H&E Staining: Haematoxylin and Eosin Staining
WT: Wildtype
KO: Knockout
SVF: Stromal vascular fraction
EDL: Extensor digitorum longus
