# Supplementary material for: Chchd10 is dispensable for myogenesis but critical for adipose browning
Source: Cell Regen. 2022 Apr 1;11:14. doi: 10.1186/s13619-022-00111-0 (PMC8975916; doi:10.1186/s13619-022-00111-0)
Supplement: Supplementary file 1 — Additional file 1. [file 13619_2022_111_MOESM1_ESM.pdf]

Supplemental Table 1. List of primers for qPCR analysis.

Gene	Primer sequence
18S	F: AGTCCCTGCCCTTTGTACACA R: CGATCCGAGGGCCTCACTA
Chchd10	F: CAGCCGGGTCTTATGGCTC R: CAGGCTCTGAATTTCCCCCAC
Ucp1	F: AGGCTTCCAGTACCATTAGGT R: CTGAGTGAGGCAAAGCTGATTT
Pgc1α	F:TATGGAGTGACATAGAGTGTGCT R: CCACTTCAATCCACCCAGAAAG
Pparγ	F:TCGCTGATGCACTGCCTATG R: GAGAGGTCCACAGAGCTGATT
Pparγ2	F: GGAAGACCACTCGCATTCCTT R: GTAATCAGCAACCATTGGGTCA
MyoD	F: GGCTACGACACCGCCTACTA R: CGACTCTGGTGGTGCATCTG
MyoG	F: TGCCCAGTGAATGCAACTCC R: TTGGGCATGGTTTCGTCTGG
Myh4	F: AGGACCAACTGAGTGAAGTGA R: GGGAAAACTCGCCTGACTCTG
Myh8	F: GGAGAGGATTGAGGCCCAAAA R: CACGGTCACTTTCCCTCCATC
Cox7a1	F: GCTCTGGTCCGGTCTTTTAGC R: GTACTGGGAGGTCATTGTCGG
Cox8b	F: GCGAAGTTCACAGTGGTTCC R: GGAACCATGAAGCCAACGAC

Supplemental Table 2. List of antibodies used in this study

Antibody	Dilution (WB)	Dilution (IF,IHC)	Manufacture
UCP1	1:2000	1:200	Abcam
AP2	1:1000		Santa Cruz
PPARγ	1:1000		Santa Cruz
PGC1α	1:1000		Santa Cruz
FABP4	1:1000		Abcam
OXPHOS	1:2000		Abcam
CHCHD10	1:500	1:200	Proteintech (CAT#25671-1-AP)
β-tublin	1:1000		Abcam
β-actin	1:1000		Santa Cruz
GAPDH	1:1000		Santa Cruz
Goat Anti-Mouse IgG(H + L)-HRP Conjugate	1:10000		Jackson Immuno Research Labs
Goat Anti-Rabbit IgG(H + L)-HRP Conjugate	1:10000		Jackson Immuno Research Labs
PAX7		1:10	Developmental Studies Hybridoma Bank
MF20	1:200	1:100	Developmental Studies Hybridoma Bank
MYOG	1:500	1:500	Developmental Studies Hybridoma Bank
TDP43	1:500	1:200	Abcam
568 goat polyclonal anti-mouse IgG1		1:1000	Thermo Fisher
488 goat polyclonal anti-rabbit IgG		1:1000	Thermo Fisher
488 goat polyclonal anti-mouse IgG2b		1:1000	Thermo Fisher
